# Lipome géant latéro-cervical chez un nourrisson de 6 mois: à propos d’un cas

**DOI:** 10.11604/pamj.2025.51.31.47251

**Published:** 2025-06-04

**Authors:** Cheikh Diene Niang, Mame Coumba Sarr, Moussa Ba, Brice Toko, Mouhamed Barry, Faty Fall, Fallou Niang, Ibou Thiam, Mame Sanou Diouf

**Affiliations:** 1 Service d'Oto-rhino-laryngologie, Centre Hospitalier National Cheikh Ahmadoul Khadim de Touba, Touba, Sénégal; 2 Service d'Oto-rhino-laryngologie, Centre Hospitalier Matlaboul Fawzeyni de Touba, Touba, Sénégal; 3 Service d'Anatomie et Cytologie Pathologique, l'Hôpital Aristide Le Dantec, Dakar, Sénégal

**Keywords:** Lipome géant, cervical, chirurgie, nourrisson, cas clinique, Giant lipoma, cervical, surgery, infant, case report

## Abstract

Les lipomes sont des néoplasmes d'origine mésenchymateuse qui intéressent la région de la tête et du cou dans 13% des cas. Cependant, ils sont rarement décrits dans la littérature chez la population pédiatrique. Nous rapportons le cas d'un nourrisson de 6 mois, sans antécédents pathologiques particuliers, admis pour la prise en charge d'une volumineuse masse latéro-cervicale gauche évoluant depuis 4 mois. L'examen oto-rhino-laryngologique (ORL) retrouvait une volumineuse masse latéro-cervicale gauche s'étendant jusqu'au niveau sous-mental d'environ 7cm de grand axe sans signe de compression. Le scanner cervical objectivait une masse de densité graisseuse, compatible avec un lipome. Le patient a bénéficié d'une cervicotomie exploratrice avec exérèse de la masse. Les résultats histologiques étaient en faveur d'un lipome. On note une évolution clinique satisfaisante sans récidive avec un recul de 15 mois. Les lipomes cervicaux, bien que rares chez la population pédiatrique, doivent être évoqués devant une tuméfaction cervicale chez le nourrisson. Sa description clinique se rapproche de celle du lymphangiome kystique.

## Abstract (en)

Lipomas are mesenchymal neoplasms that affect the head and neck region in about 13% of cases. However, they are rarely reported in the pediatric population. We here report the case of a 6-month-old infant with no notable medical history, admitted for the management of a large left lateral cervical mass that had been progressing for 4 months. Ear, nose and throat (ENT) examination revealed a large left lateral cervical mass extending to the submental area, approximately 7cm in length, with no signs of compression. Cervical CT scan showed a fat-density mass, consistent with a lipoma. The patient underwent exploratory cervicotomy with excision of the mass. Histological analysis confirmed the diagnosis of lipoma. The clinical outcome was favorable, with no recurrence after 15 months of follow-up. Although rare in the pediatric population, cervical lipomas should be considered in infants with a cervical swelling. Its clinical manifestation is similar to that of a cystic lymphangioma.

## Introduction

Les lipomes sont des tumeurs bénignes mésenchymateuses caractérisées par la prolifération d'adipocytes matures [1]. C'est le néoplasme le plus courant des tissus mous chez l'adulte. Cependant, ils sont rares chez le nourrisson [2]. Les sites les plus courants sont le tronc et les membres. Moins de 15% des lipomes surviennent dans la tête et le cou [3]. Sur le plan épidémiologique, les lipomes ont tendance à apparaître au cours des cinquième et sixième décennies de la vie [2]. Nous rapportons ici un cas rare de lipome géant cervical chez un nourrisson de 6 mois.

## Patient et observation

**Informations destinées au patient:** nous rapportons le cas d'un nourrisson de 6 mois, reçu en consultation pour la prise en charge d'une volumineuse masse latéro-cervicale gauche évoluant depuis 4 mois sans signes de compression ni fièvre associée. Aucun antécédent pathologique particulier n'a été rapporté.

**Figure 1:**
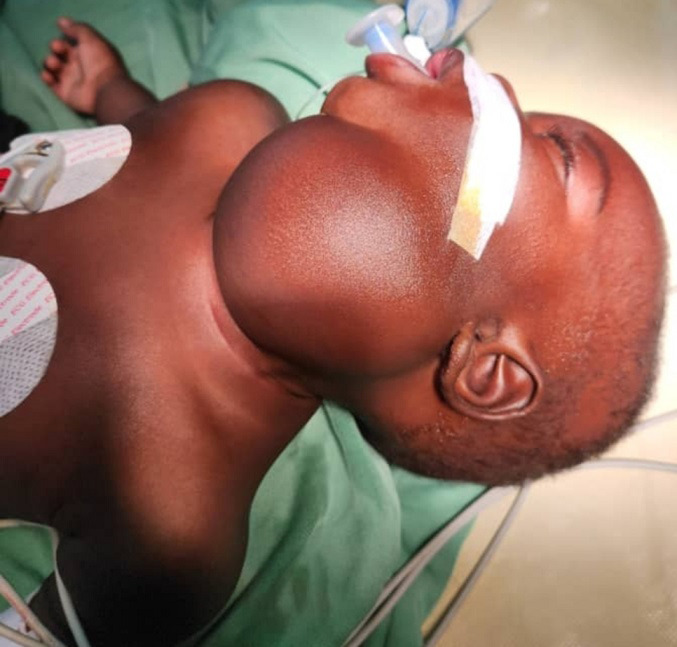
image préopératoire du patient

**Résultats cliniques:** l'examen ORL retrouvait une volumineuse masse sous-mentale s'étendant jusqu'au niveau latéro-cervical gauche (Figure 1), molle, mobile par rapport aux deux plans, d'environ 7cm de grand axe, sans adénopathies associées.

**Figure 2:**
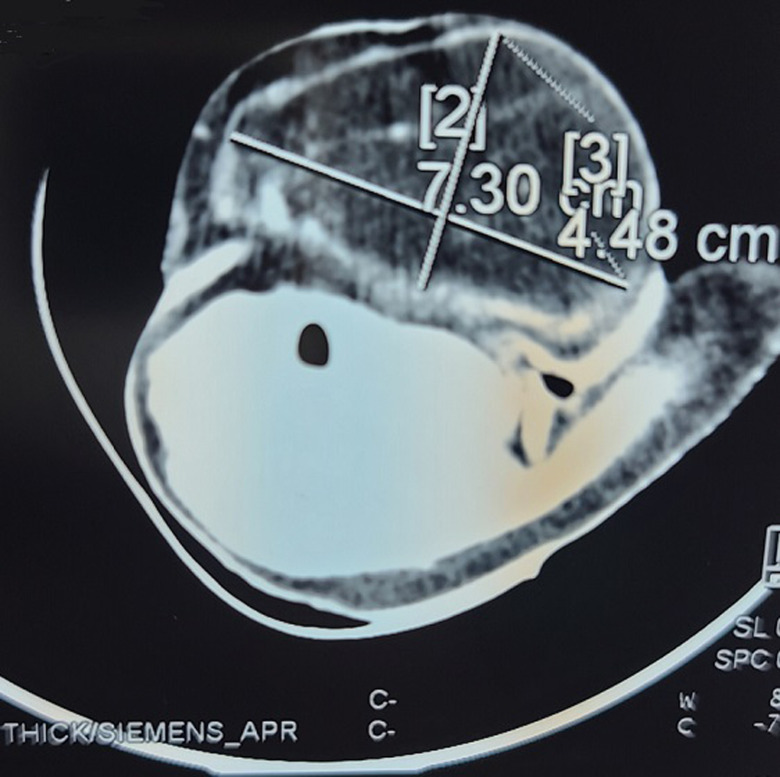
tomodensitométrie cervicale coupe axiale

**Démarche diagnostique:** l'échographie montre une masse décrite comme une structure tissulaire sous-cutanée d'allure graisseuse de 35mm d'épaisseur sur 63mm de grand diamètre. Elle est homogène, bien limitée avec des contours réguliers, faiblement vascularisée au Doppler couleur. Le scanner cervical objectivait une masse de densité graisseuse, sous-cutanée latéro-cervicale gauche de 73x45mm de diamètre dans le plan axial sur 35mm dans le plan sagittal, compatible avec un lipome (Figure 2).

**Figure 3:**
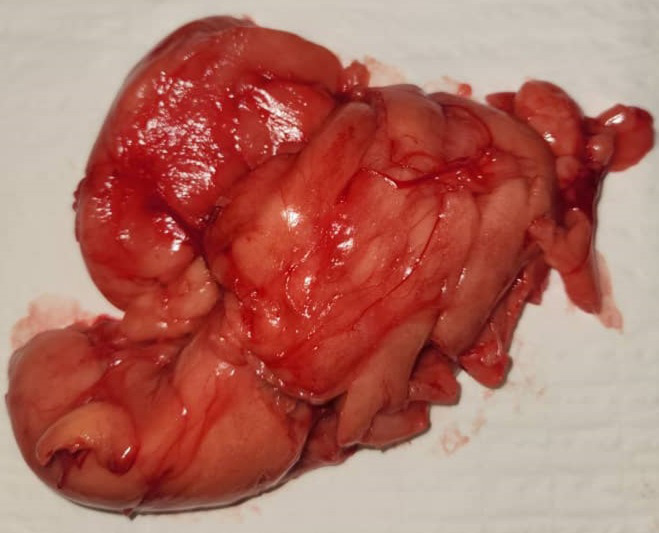
pièce opératoire

**Intervention thérapeutique:** le patient a bénéficié d'une cervicotomie exploratrice retrouvant une volumineuse masse graisseuse pseudo-encapsulée en contact avec les gros vaisseaux. L'exérèse complète de la masse fut réalisée (Figure 3). Les suites opératoires étaient simples. Les résultats histologiques de la pièce opératoire étaient en faveur d'un lipome.

**Suivi et résultats:** on note une bonne évolution clinique sans récidive avec un recul de 22 mois.

**Consentement éclairé:** les parents de notre patient ont donné leur consentement écrit pour la publication de ses informations cliniques et de toute image d'identification.

## Discussion

Les lipomes sont des tumeurs bénignes d'origine mésenchymateuse qui se développent lentement [4]. Ils sont généralement asymptomatiques, à moins qu'ils n'affectent ou ne compriment les structures environnantes [5,6]. Les lipomes de la tête et du cou chez l'enfant sont rares et leur présentation peut être atypique par rapport à celle de l'adulte [2,5]. Le site le plus courant dans la région de la tête et du cou est le triangle postérieur cervical [3,7].

Le diagnostic des lipomes de la tête et du cou commence par un bon examen clinique. Les lipomes sont des masses mobiles non douloureuses, généralement rondes, avec une sensation caractéristique de douceur et de pâte à la palpation, la peau qui les recouvre étant souvent froide en raison de la qualité isolante de la graisse [6]. Ces masses peuvent engendrer uniquement une gêne esthétique, mais certains lipomes peuvent induire des signes de compression des voies aérodigestives supérieures ou être suspects de transformation maligne [6].

Notre cas se présentait comme une masse cervicale asymptomatique, ayant fait évoquer en premier lieu un lymphangiome kystique [5]. Vu l'âge et la présentation clinique, illustrant ainsi l'importance des imageries préopératoires pour éliminer les diagnostics différentiels et guider la prise en charge chirurgicale. Une échographie peut donner un diagnostic clair et rapide d'un lipome. A l'échographie, un lipome est peu caractéristique (hypo-, iso- ou hyperéchogène). A la tomodensitométrie, le lipome cervical est de densité graisseuse, bien délimité, ne prenant pas le contraste, permettant de le différencier d'un liposarcome plus hétérogène et à contours irréguliers. A l'imagerie par résonance magnétique, la graisse se caractérise par un hypersignal en T1 et un hyposignal en T2, avec un bon contraste après injection [6,8].

Le traitement du lipome cervical est l'exérèse chirurgicale, qui doit être complète sous peine de récidives ou d'une dégénérescence maligne [6]. La lipoaspiration constitue une alternative intéressante en raison des résultats. Cependant, avec un risque de récidive plus important par rapport aux techniques chirurgicales conventionnelles [2].

Une surveillance postopératoire régulière et prolongée est de mise en raison du risque de récidive et de transformation du lipome géant en liposarcome [8].

## Conclusion

Les lipomes géants cervicaux sont rares chez le nourrisson. Les examens d'imagerie doivent être réalisés devant toute masse cervicale indolore et permettent d'éliminer la plupart des diagnostics différentiels, notamment le lymphangiome kystique. Le traitement repose essentiellement sur l'exérèse chirurgicale complète de la masse pour éviter la récidive, et le suivi postopératoire doit être prolongé.
